# A mixed-methods study assessing the performance of a clinical decision support tool for *Clostridioides difficile* testing for patients receiving laxatives

**DOI:** 10.1017/ice.2025.30

**Published:** 2025-05

**Authors:** David R. Peaper, Shardul N. Rathod, L. Scott Sussman, Marwan M. Azar, Christina Murdzek, Scott C. Roberts, Eric M. Tichy, Jeffrey E. Topal, Nitu Kashyap, Dayna McManus, Richard A. Martinello

**Affiliations:** 1 Department of Laboratory Medicine, Yale School of Medicine, New Haven, CT, USA; 2 Department of Epidemiology of Microbial Diseases, Yale School of Public Health, New Haven, CT, USA; 3 Department of Healthcare Epidemiology and Infection Prevention, Northwestern Memorial Hospital, Chicago, IL, USA; 4 Department of Internal Medicine, Yale School of Medicine, New Haven, CT, USA; 5 Department of Infection Prevention, Yale New Haven Health, New Haven, CT, USA; 6 Supply Chain Management, Mayo Clinic, Rochester, MN, USA; 7 Department of Pharmacy Services, Yale New Haven Hospital, New Haven, CT, USA; 8 Yale New Haven Health, Yale School of Medicine, New Haven, CT, USA; 9 Department of Pediatrics, Yale School of Medicine, New Haven, CT, USA

## Abstract

**Objective:**

To better understand clinicians’ rationale for ordering testing for *C. difficile* infection (CDI) for patients receiving laxatives and the impact of the implementation of a clinical decision support (CDS) intervention.

**Design:**

A mixed-methods, case series was performed from March 2, 2017 to December 31, 2018.

**Setting:**

Yale New Haven Hospital, a 1,541 bed tertiary academic medical center.

**Participants:**

Hospitalized patients ≥ 18 years old, and clinicians who were alerted by the CDS.

**Intervention:**

CDS was triggered in real-time when a clinician sought to order testing for CDI for a patient who received one or more doses of laxatives within the preceding 24 hours.

**Results:**

A total of 3,376 CDS alerts were triggered during the 21-month study period from 2,567 unique clinician interactions. Clinicians bypassed the CDS alert 74.5% of the time, more frequent among residents (48.3% bypass vs. 39.9% accept) and advanced practice providers (APPs) (34.9% bypass vs. 30.6% accept) than attendings (11.3% bypass vs. 22.5% accept). Ordering clinicians noted increased stool frequency/output (48%), current antibiotic exposure (34%), and instructions by an attending physician to test (28%) were among the most common reasons for overriding the alert and proceeding with testing for CDI.

**Conclusions:**

Testing for CDI despite patient laxative use was associated with an increased clinician concern for CDI, patient risk for CDI, and attending physician instruction for testing. Attendings frequently accepted CDS guidance while residents and APPs often reinstated CDI test orders, suggesting a need for greater empowerment and discretion when ordering tests.

## Background

*Clostridioides difficile* infection (CDI) is one of the most common healthcare-associated infections in the United States^1–4^ and leads to an estimated 14,000 to 20,000 deaths in the US each year.^5^ The cost of CDI ranges from $3,000 to greater than $24,000 per patient in acute-care hospitals.^5–7^ CDI is defined as the presence of ≥ 3 unformed stools within 24 hours in addition to laboratory evidence of toxigenic *C. difficile* in stool.^8,9^ While most cases of hospital-onset diarrhea are thought to be noninfectious, studies have attributed 10%–20% of cases of hospital-onset diarrhea to CDI.^10–12^

A common cause of hospital-onset diarrhea is the use of laxatives.^11,13,14^ As many as 44% of patients tested for CDI received laxatives within 48 hours prior to testing.^15,16^ As tests currently used to detect CDI identify the presence of the pathogen and its ability to produce toxins rather than the true presence of CDI, clinicians must carefully choose when to test for CDI. This includes nucleic acid amplification tests (NAATs) for *C. difficile* which do not differentiate between colonization and CDI if testing criteria are not followed.^17,18^ Increases in reported CDI rates in recent years may be associated with widespread use of NAATs, compounded by inappropriate *C. difficile* testing practices.^19–21^

There are limited data examining the association between laxative use and *C. difficile* testing, though laxative use does not preclude CDI diagnosis.^22,23^ However, *C. difficile* testing in this population may increase the likelihood of identifying patients with laxative-related diarrhea and asymptomatic *C. difficile* colonization, leading to misclassification and subsequent unnecessary treatment for CDI.^24,25^ Clinical decision support (CDS) alerts are common interventions to guide appropriate testing including for *C. difficile*.^26,27^ Previously reported CDS alerts for *C. difficile* testing have notified clinicians of laxative use within 24, 48, or 72 hours prior to testing.^28,29^

Our academic medical center implemented a CDS alert called a “Best Practice Advisory” (BPA) to notify clinicians of laxative administration within 24 hours prior to *C. difficile* testing. We first assessed clinician behavior when interacting with the alert to determine its effectiveness in reducing *C. difficile* testing among patients receiving laxatives. We then assessed clinician rationale for overriding the alert to proceed with *C. difficile* testing despite recent laxative administration.

## Methods

### Study design and participants

We conducted a mixed-methods, case series study from March 2, 2017 to December 31, 2018 at Yale New Haven Hospital (YNHH), a 1,541-bed tertiary care academic medical center located in New Haven, Connecticut. This study period encompasses the time frame since the implementation of the CDI laxative CDS alert and includes all instances in which the CDS alert was presented to clinicians for inpatients ≥ 18 years.

### Clinical decision support

CDS was designed as an interactive BPA. The BPA presented as a “pop-up window” containing language to alert and guide the clinician when they are ordering testing for CDI if a laxative had been administered to the patient during the past 24 hours. This BPA (Epic^®^ Systems Corporation, Verona, WI) (eFigure 1) was presented in real time to the ordering clinician and offered the option to either a default selection to “Remove” (i.e., cancel) or “Keep” the order for *C. difficile* testing, and these actions were considered as “Acceptance” or “Override” of the BPA, respectively. To continue with ordering testing for CDI, the BPA requires the clinician to purposefully select “Keep.” Otherwise, the action to “Remove” the order is the default choice, and the order for testing for CDI would be canceled when the clinician closed the BPA. Clinicians were educated about the importance of considering laxatives as a cause of healthcare-associated diarrhea and the BPA implementation by presentations in multiple conference settings and by email.

### Laboratory methods

Testing for *C. difficile* was performed using a two-stage algorithm. The first test was a combination of *C. difficile*-specific glutamate dehydrogenase (GDH)/Toxin A/B immunoassay (Quik Check Complete, Techlab Inc., Blacksburg, VA). Samples with discordant GDH and toxin results (i.e., one positive, the other negative) were reflexively tested by cytotoxin neutralization.^30^ Results of cytotoxin neutralization testing were considered the final result in those cases. Formed stools not conforming to the shape of the container were rejected by the laboratory.

### Data collection

Data were extracted from the electronic health record (EHR) for all instances of the BPA during the study period. Demographics collected included clinician type, alert date and time, admission data, and patient location. Pharmacy data included the laxative administered most closely to the time of *C. difficile* ordering, administration of a high-risk antibiotic, and administration of a proton-pump inhibitor (PPI). Laboratory data included order date and time, ordering clinician, patient location, and laboratory test results. *C. difficile* laboratory data was also extracted from the Epic^®^ “Beaker” laboratory information system to verify the initial data extract and determine if additional *C. difficile* testing was performed unrelated to the CDS alert.

### Classification of clinician interactions

While most clinicians interacted with the BPA one time and either accepted the guidance and discontinued testing for CDI, or overrode guidance and proceeded with testing, some clinicians quickly initiated a new order for testing after their initial acceptance of the BPA led to discontinuation of the CDI test order. For example, a provider “Accepting,” “Accepting,” and then “Overriding” within the span of five minutes would have a 66.6% “Acceptance” rate but ultimately yield an order for *C. difficile* testing. We considered all alerts to a single provider for a single patient within the span of five minutes as an “interaction,” and we counted the number of individual alerts presented to a provider for each interaction. The clinician information and alert date/time stamp were used to classify interactions as either accept or override based on the outcome after five minutes time from the first BPA interaction.

### Definitions

A high-risk antibiotic exposure was defined as use of ≥ 1 of the following: cefepime, ceftazidime, clindamycin, ciprofloxacin, ertapenem, meropenem, moxifloxacin, or piperacillin/tazobactam within seven days prior to *C. difficile* testing. PPI use was similarly defined as exposure within seven days prior to *C. difficile* testing.

Hospital Service was assigned based on order location consistent with the units’ Center for Disease Control and Prevention, National Healthcare Safety Network mapping. Physician Assistants and Advanced Practice Registered Nurses were grouped together in the Advanced Practice Provider (APP) category. Since rates of BPA acceptance did not significantly differ among patients receiving docusate, magnesium citrate, bisacodyl, or polyethylene glycol, these were consolidated into an “Other” category for triggering laxative medications. BPA were considered presented to the primary hospital team if they occurred between 7:00 AM and 7:00 PM Monday to Friday. BPA outside of these times were classified as being presented to “Cross Coverage” clinicians.

### Direct assessment of clinician behavior

An investigator’s (SNR) mobile device was linked to the EHR to receive alerts when the BPA was triggered from August to December 2018 and the clinician chose to “Keep/Accept” and proceed in ordering testing for *C. difficile*. During a time-based period of convenience, the ordering clinician was telephoned in real-time to explore the clinical rationale for testing for CDI in patients receiving laxatives. A brief, standardized series of open-ended questions was used to guide the discussion, and clinician responses were subsequently coded into themes following a grounded theory approach by SNR and RAM. Qualitative data collection was concluded after reaching a point of saturation.

### Statistical analysis

Data analysis was performed using SPSS (IBM^®^, Chicago IL). Statistical significance was determined using a p-value of < 0.05. Statistical tests used are indicated in table and figure legends. Results of single factor analysis were used to generate a binomial regression analysis model in SPSS to identify factors significantly associated with alert rejection. GraphPad Prism (GraphPad^®^ Software, Boston MA) was used to generate Fig. 2.

### Institutional review board approval

The Institutional Review Board of Yale University deemed the study as exempt from approval, designating the study as a quality improvement project.

## Results

**Figure 1.**
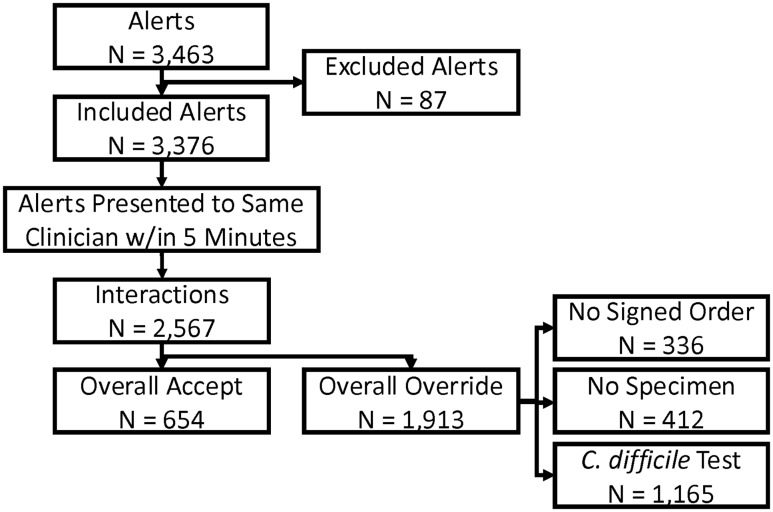
Flowchart of CDS alerts, clinician actions, and testing for *C. difficile*.

During the 21-month study period, there were 9,844 samples tested for *C. difficile*, and 707 (8.2%) were positive. The BPA fired 3,376 times for 1,849 unique patients from 1,970 patient encounters, and 42.3% of all BPA were accepted (Fig. 1), which resulted in an initial cancelation of testing for CDI. Often, a clinician would accept the BPA, proceed to re-order *C. difficile* testing, and then override the subsequent BPA within one minute. When the BPA was classified based on clinician interactions for a care episode, there were 2,567 unique interactions and the BPA acceptance rate was 25.4% (Fig. 1). Most interactions within five minutes consisted of a single alert (n=1,911), but interactions with two alerts (n=545) or more (n=111) were common. Among the 1,913 overridden BPA, 1,165 tests (60.9%) for *C. difficile* were completed.

**Figure 2.**
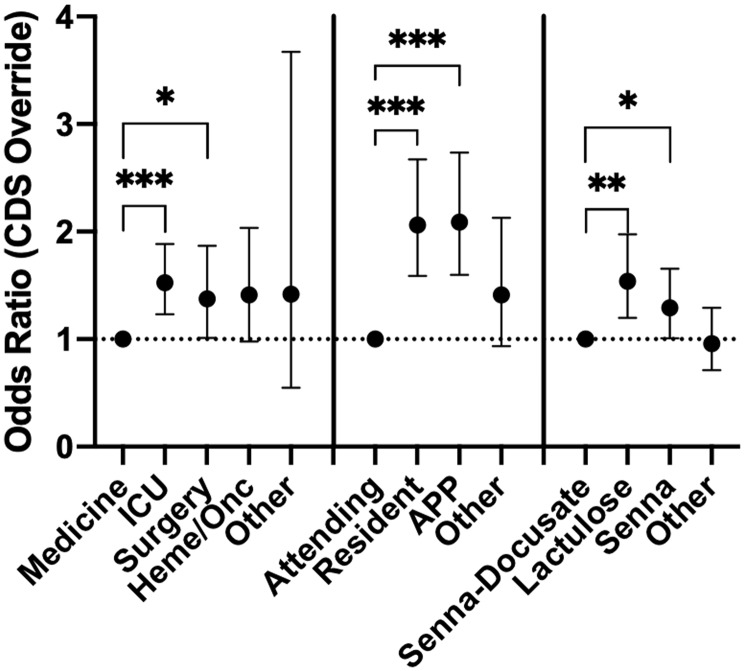
Odds ratio measures for overriding CDS alert and ordering testing for *C. difficile* for factors identified as significant by regression analysis. Statistical significance between the indicated factors is denoted as follows: * < 0.05; ** < 0.01, *** < 0.001.

Rates of BPA acceptance varied with respect to patient age, sex, duration of admission, hospital service, clinician type, alert medication, and number of alerts per interaction (Table 1; Fig. 2). There was no significant correlation shown between cross-coverage care, history of CDI 7–90 days prior to the BPA, exposure to high-risk antibiotics or PPIs, and BPA acceptance (Table 1).

**Table 1.** Patient demographic risk factors for *C. difficile* clinical decision support (CDS) override

	CDS Accept	CDS Override	p-value
**Total Interactions, n**	654	1913	
Age, mean (SD)	65.2 (15.9)	63.4 (16.1)	0.025
Days of Admission, mean (SD)	12.2 (23.9)	12.8 (23.0)	0.011
Male, n (%)	313 (47.9%)	843 (44.1%)	0.045
Cross Coverage, n (%)	306 (48.0)	852 (44.1)	0.101
**Hospital Service**			
Medicine (General), n (%)	352 (53.8%)	798 (41.7%)	< 0.001
ICU, n (%)	183 (28.0%)	720 (37.6%)	
Surgery (General), n (%)	69 (10.6%)	223 (11.7%)	
Hematology/Oncology, n (%)	44 (6.7%)	154 (8.1%)	
Other, n (%)	6 (0.9)	18 (0.9)	
**Clinician Type**			
Attending Physician, n (%)	147 (22.5%)	216 (11.3%)	< 0.001
Resident, n (%)	261 (39.9%)	924 (48.3%)	
APP, n (%)	200 (30.6%)	668 (34.9%)	
Other, n (%)	46 (7.0%)	105 (5.5%)	
**Alert Laxative**			
Senna-docusate, n (%)	355 (54.3%)	904 (47.3%)	0.001
Lactulose, n (%)	111 (17.0%)	433 (22.6%)	
Senna, n (%)	112 (17.1%)	388 (20.3%)	
Other, n (%)	76 (11.6%)	188 (9.8%)	
**Interaction Summary**			
1 Alert for Interaction, n (%)	555 (84.9%)	1356 (70.9%)	< 0.001
2 Alerts for Interaction, n (%)	87 (13.3%)	458 (23.9%)	
≥ 3 Alerts for Interaction, n (%)	12 (1.8%)	99 (5.2%)	
**Risk Factors**			
*C. diff* Pos. 7 – 90 Days Prior, n (%)	8 (1.3%)	38 (2.0%)	0.235
High-Risk Antibiotic Use, n (%)	381 (58.3%)	1177 (61.5%)	0.151
PPI Use, n (%)	248 (37.9%)	741 (38.7%)	0.745

CDI test orders from the Medicine service, those entered by attending physicians, and for patients receiving senna-docusate were associated with being significantly more likely to accept the BPA (Table 1; Fig. 2). ICU service care, CDI test orders entered by a resident or advanced practice provider, and the receipt of lactulose were significantly more likely to lead to BPA override (Table 1; Fig. 2).

**Figure 3.**
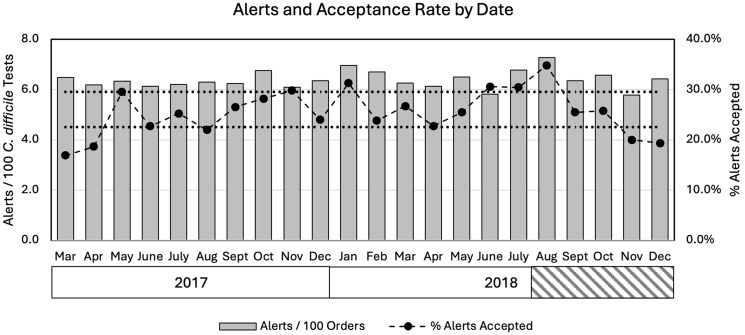
Frequency of CDS alerts and their rate of acceptance over time. The median acceptance rate was 25.5%. The dotted lines indicate the interquartile range for overall CDS alert acceptance. The shaded box in 2018 indicates the period of time when phone calls were made to ordering clinicians.

In contrast with the initial contingency table analysis, the binomial regression model showed that age, sex, and duration of admission were not significantly associated with BPA override (eTable 1). Hospital service, the receipt of lactulose or senna as the laxative triggering the BPA alert, and ordering clinician type remained significantly associated with BPA override. The relationships between the BPA override for each variable compared to the class reference are shown (Fig. 2). There was a mean of 117 alerts per month, and no temporal patterns emerged when data were reviewed by month or provider type (Fig. 3, eFigure 2).

Of the specimens submitted for testing from patients having received laxatives, 8.6% were positive for *C. difficile* toxin either on the rapid assay (4.1%) or cytotoxin neutralization (4.5%).

A convenience sample of 100 clinicians who overrode the BPA and ordered CDI testing for patients receiving laxatives were telephoned in real-time and all completed the interview (eTable 2). Three primary themes emerged from clinician responses for overriding the BPA: 1) concern that a patient was considered high-risk for CDI, 2) concern that a patient was presenting with severe manifestations of CDI, and 3) instructions by an attending physician to test (Table 2). Increased stool frequency/output and current antibiotic exposure were among the most common reasons for clinicians overriding the BPA (48% and 34%, respectively).

**Table 2.** Themes associated with clinical decision support alert override based on 100 provider phone calls

Theme	N	Representative Quotes
**Patient considered as high-risk for CDI**		
Subtheme 1: High Risk Not Specified	38	“He was at risk for multiple organ failure and shock. We thought the risk of missing C. diff was higher than the risk of getting a false positive.”
Subtheme 2: Current Antibiotic Exposure	34	“Although she was on laxatives PRN, she has been on Vancomycin, Zosyn, and Ciprofloxacin for the past two weeks.”
**Patient presenting with more severe manifestations of suspected CDI**		
Subtheme 1: Increase Stool Frequency/Output	48	“He had 7 loose stools last night and another this morning. We were thinking that if he had one more loose stool this morning, we would send in a test.”
Subtheme 2: Increase White Blood Cell Count	20	“Her white blood cell count is trending upwards, and we’re unsure of its source.”
**Clinician being instructed by an attending physician to order testing**	28	“My attending, a critical care physician, feels strongly about there being a high pre-test probability of *C. diff*.”

Finally, we sought to ascertain whether rates of CDI test result positivity varied among patients who a) had received laxatives and had testing performed despite the BPA, b) had testing performed within 14 days of BPA acceptance or in which an override did not generate a valid test, and c) were in the general population tested during the study period who had not received laxatives (eTables 3 and 4). There was no significant difference in positive rates of CDI testing between testing ordered immediately after the BPA and testing unrelated to a BPA (eTable 5).

## Discussion

CDS alerts are common interventions to guide appropriate testing for *C. difficile*. The BPA was initially accepted in 42.3% of the interactions. However, within a 5-minute window, this acceptance decreased to 25.4%. This overall rate of acceptance is consistent with that reported for “soft-stop” BPA (i.e., an intervention that allows the clinician to proceed with the original intent, if desired) at other academic medical centers, ranging from 25 to 32%.^31^ Interestingly, the rate of BPA acceptance was lowest at the time of implementation despite extensive prior clinician education.

We found that attending clinicians were much more likely to accept the BPA, which aligns with the qualitative survey data and current literature.^32,33^ APPs and residents often cited attending instructions to test for *C. difficile* as the rationale for overriding the BPA. It is possible that the patients’ receipt of laxatives was not relayed back to the attending for further discussion and consideration. In contrast, when attending clinicians were made aware of a patient’s recent receipt of laxatives, they were much more likely to delay testing by accepting the BPA.

Interestingly, factors associated with development of CDI including previous CDI and exposure to high-risk antibiotics were not significantly associated with overriding the CDS alert. We further hypothesized that coverage by the non-primary team during off hours would lead to higher rates of alert override, though this was not identified. Although related to the development of CDI, some of these factors were not associated with clinician decision-making by themselves with respect to overriding the laxative alert.

Given our institution’s two-step assay of cytotoxin neutralization to adjudicate discordant rapid test results, these non-NAAT methods contributed to a conservative approach with the BPA alert calibrated to minimize risk of missing the opportunity to test a patient who truly has a CDI by using a 24-hour laxative window and a soft-stop rather than hard-stop for *C. difficile* test ordering. Our toxin positivity rates for the rapid assay (4.1%) and cytotoxin neutralization assay (4.5%) are reflective of the conservative approach. These rates of positivity are lower than those reported for institutions using PCR to diagnose CDI with use of a similar BPA alert.^28^

The qualitative assessment revealed that there were three major themes provided for overriding the CDS alert. Many clinicians were concerned that their patient was either at high risk for CDI or the patient was felt to display signs of severe CDI. Patients in ICU settings have the greatest risk for severe CDI due to their underlying medical conditions, drug exposures, and risk for adverse outcomes^34–37^; location in the ICU was the second most common location for CDS alerts. However, the number of alerts that generated phone calls for ICU patients were not substantially different from the general population receiving alerts. While some clinicians who were called indicated that they would reconsider CDI testing at that time, the rate of completed tests did not significantly differ between interactions with a phone call (56% completed testing) and those without a call (60% completed testing; data not shown). Interestingly, the third major reason for overriding the BPA was due to instructions from an attending physician to order a test. This highlights the importance of incorporating the relationship between clinician type and BPA override in educational materials to empower residents and APPs. An emphasis on tailored resident/APP education may improve the level of collaboration between clinician types to reconsider care plans in the context of CDI diagnostic stewardship. This finding may be significant in bridging persistent gaps in understanding CDI testing practices in patients receiving laxatives and may be the target of future qualitative studies and institutional quality improvement initiatives.

Alert fatigue is well documented.^38,39^ At times in our study, the ordering clinician first became aware of laxative use after receiving a phone call, despite having just bypassed the CDS alert informing them. It is likely that many clinicians dismissed the alert without fully evaluating its contents.^40^ While overall rates of testing did not differ between the interactions that received a phone call and those that did not, there were clinicians who may not have pursued *C. difficile* testing based on the phone conversation. Future projects should investigate less intensive methods than phone calls to raise clinician awareness of decision support guidance.

Our study has limitations. It relied upon the information contained in the EHR and laboratory information system, so clinician decision-making could not be fully interrogated. Residents were the largest clinician group in our study, but we did not track year-in-training when the alert was fired. It would be interesting to determine if more senior residents responded differently than interns. Confounding effects are possible in our data regarding hospital service, care team structure, and administered laxatives. With respect to the phone call data, interactions generating a phone call were based upon convenience and not randomly selected. However, there was a reasonable distribution of time of day and evening, patient location, clinician role, and laxative administered among the interactions with phone calls.

Optimal diagnostic stewardship is necessary to reduce CDI incidence rates. The implementation of a BPA alert at our institution was moderately effective at discouraging *C. difficile* testing in patients receiving laxatives. Clinicians often overrode this BPA when they perceived that their patient was at high risk for CDI, had more severe manifestations of a potential CDI, or were instructed to test by an attending physician. Incorporation of the relationship between clinician type and BPA override into educational materials may further empower residents and APPs to reconsider care plans in the context of CDI diagnostic stewardship.

## Supporting information

Peaper et al. supplementary material Peaper et al. supplementary material

## Supplementary material

The supplementary material for this article can be found at http://doi.org/10.1017/ice.2025.30.
