# Supplementary material for: A mixed-methods study assessing the performance of a clinical decision support tool for Clostridioides difficile testing for patients receiving laxatives
Source: Infect Control Hosp Epidemiol. 2025 Mar 13;46(5):497–503. doi: 10.1017/ice.2025.30 (PMC12034449; doi:10.1017/ice.2025.30)
Supplement: Peaper et al. supplementary material [file S0899823X25000303sup001.docx]

**Supplemental Tables**

**eTable 1. Binomial linear regression analysis to assess factors associated with alert override by bivariate analyses.**

|  | Adjusted Odds Ratio | Lower 95%tile CI | Upper 95%tile CI | p-value |
| --- | --- | --- | --- | --- |
| Hospital Service (Ref = Medicine Unit) |  |  |  | 0.002 |
| ICU v. Medicine Unit | 1.524 | 1.233 | 1.884 | <0.001 |
| Surgery v. Medicine Unit | 1.374 | 1.01 | 1.869 | 0.043 |
| Heme/Onc v. Medicine Unit | 1.411 | 0.978 | 2.035 | 0.065 |
| Other v. Medicine Unit | 1.420 | 0.549 | 3.672 | 0.470 |
| Provider Type (Ref = Attending) |  |  |  | <0.001 |
| Resident v. Attending | 2.060 | 1.588 | 2.673 | <0.001 |
| App v. Attending | 2.090 | 1.598 | 2.734 | <0.001 |
| Other v. Attending | 1.412 | 0.936 | 2.128 | 0.100 |
| Alert Medication (Ref = Senna-Docusate(S-D)) |  |  |  | 0.002 |
| Lactulose v. S-D | 1.538 | 1.197 | 1.976 | 0.001 |
| Senna v. S-D | 1.293 | 1.009 | 1.656 | 0.042 |
| Other v. S-D | 0.958 | 0.711 | 1.292 | 0.781 |

**eTable 2: Interview to assess rationale for bypassing BPA**

|  | Question |
| --- | --- |
| 1 | I see that [Patient Name] has received laxatives in the past 24 hours. Could you elaborate as to why the *C. difficile* test was ordered? |
| 2 | Did you consider stopping the laxatives and reassessing, first? If so, why did you proceed to order the test? |
| 3 | How were you made aware that the patient has diarrhea? (Ex: nurse, patient chart, patient) |

**eTable 3: Results of *C. difficile* testing for specimens submitted where CDS laxative alert was or was not present.**

|  | Negative | Positive | |
| --- | --- | --- | --- |
| CDS Laxative Alert | 1080 (91.4%) | 101 (8.6%) | |
| No CDS Laxative Alert | 7956 (91.8%) | 707 (8.2%) | |
| Chi-Square analysis was not significant (p = 0.651) | | |  |

**eTable 4: Comparison of results of subsequent specimens submitted for *C. difficile* testing within 14 days of alert and specimens from patients who had not received laxatives within 24 hours of test order.**

|  | Negative | Positive |
| --- | --- | --- |
| Specimen within 14 days of an alert | 350 (91.4%) | 33 (8.6%) |
| All other specimens | 8686 (91.8%) | 775 (8.2%) |
| Chi-Square analysis was not significant (p = 0.775) | | |

**eTable 5: Results of the components of *C. difficile* testing**

|  | Rapid GDH | Rapid Toxin | Cytotoxin | Alert Specimen | Not Alert Specimen | Grand Total |
| --- | --- | --- | --- | --- | --- | --- |
| Toxigenic *C. difficile* NEG | Negative | Negative | Not Indicated | 994 | 7189 | 8183 |
|  | Positive | Negative | Negative | 86 | 765 | 851 |
| Toxigenic *C. difficile* POS | Positive | Negative | Positive | 53 | 379 | 432 |
|  | Positive | Positive | Not Indicated | 48 | 326 | 374 |
|  | Grand Total | | | 1181 | 8663 | 9840* |
| *indicates 4 patients with GDH = Neg; Tox = Pos that were reflexed to cytotoxin not included in the table. Of these 4, 2 were cytotoxin positive and 2 were cytotoxin negative. Chi-square analysis was non-significant (p = 0.343). | | | | | | |

**Supplemental Figure Legends**

**eFigure 1:** Clinical decision support “Best Practice Advisory” presented to providers when ordering *Clostridioides difficile* testing for patients who received a laxative in the preceding 24 hours. To “Remove” the order was considered “accepting” the alert, but to “Keep” the order was consider “overriding” the alert.

**eFigure 2:** Alerts and acceptance rate by provider type over time.
